# Measuring what matters: governance challenges in defining follow-up priorities in complex healthcare systems

**DOI:** 10.1186/s12961-026-01550-w

**Published:** 2026-10-28

**Authors:** Theresa Larsen, Ewa Wikström, Helle Wijk, Richard Sawatzky, Axel Wolf

**Affiliations:** 1 The Gothenburg Region, Gothenburg, Sweden; 2 Institute of Health and Care Sciences, University of Gothenburg, Gothenburg, Sweden; 3 University of Gothenburg Centre for Person-Centred Care, Sahlgrenska Academy, Gothenburg, Sweden; 4 Centre for a Dementia Friendly Society with a Person-Centred Mindset, University of Gothenburg, Gothenburg, Sweden; 5 School of Nursing, Trinity Western University, Langley, BC Canada; 6 Centre for Advancing Health Outcomes, Providence Health Care, Vancouver, BC Canada; 7 Department of Architecture and Civil Engineering, Chalmers University of Technology, Gothenburg, Sweden; 8 Department of Quality Assurance, Sahlgrenska University Hospital, Gothenburg, Sweden; 9 Centre for Health Governance, Department of Management and Organization, The School of Business, Economics and Law, University of Gothenburg, Gothenburg, Sweden

**Keywords:** Complex healthcare systems, Performance measurement, Person-centred care, Integrated care, Workshops, Health system reform, Health governance

## Abstract

**Background:**

Performance measurement plays a central role in governing healthcare system reforms, yet defining what should be monitored is often challenging in complex and decentralized systems. Rather than functioning as neutral tools, performance indicators shape how reform priorities become visible and governable within reform processes. While previous research has examined the design and effects of performance measurement systems, less is known about how follow-up priorities are negotiated when broad reform ambitions are translated into local governance. This study explores how municipal stakeholders negotiate what should become measurable during the implementation of a large-scale healthcare reform.

**Methods:**

An interpretive descriptive approach was used. Data were generated through nine stakeholder workshops involving 191 participants across municipalities in Västra Götaland County, Sweden and analysed using an abductive thematic analysis. Participants included politicians, managers, civil servants and healthcare and social care professionals. The workshops explored existing indicators, information gaps and the conditions required for meaningful follow-up of the ongoing transition towards Close Care, a national reform aiming to strengthen primary care and develop more person-centred services.

**Results:**

Four closely related empirical patterns were identified and, through abductive interpretation, synthesized into a conceptual framework comprising normative, conceptual, infrastructural and epistemic conditions shaping the feasibility of measurement-based governance. First, stakeholders negotiated why, for whom and how measurement should be used, reflecting tensions between accountability, quality improvement and learning. Second, conceptual ambiguity regarding the reform’s governing object made it difficult to determine what should be measured. Third, fragmented data infrastructures limited the availability and comparability of indicators. Fourth, limited epistemic capacity constrained how available data could be understood and used for governance and improvement.

**Conclusions:**

Rather than viewing tensions between reform ambitions and measurement feasibility as implementation failures or resistance to measurement, the findings suggest that such tensions are inherent features of governing complex healthcare systems. By examining the governance processes through which “what matters” is negotiated before becoming measurable, the study highlights measurement itself as a central governance process. Before performance measurement can guide governance, organizations must establish the normative, conceptual, infrastructural and epistemic conditions that enable measurement to become meaningful, actionable and ultimately performative.

## Background

International research on performance measurement in health and welfare organizations highlights a persistent tension between the use of indicators for quality improvement and learning versus their use for accountability and control [1, 2]. While classic frameworks such as Donabedian’s structure–process–outcome model continue to inform indicator selection, empirical studies show that the design and use of measures are shaped as much by politics and institutional logics as by technical criteria [3, 4]. Systems oriented towards external accountability may produce unintended consequences such as gaming, tunnel vision and short-termism, potentially undermining learning and improvement [2, 5]. Organizational learning theory, particularly the distinction between single- and double-loop learning [6], has been used to explain why some measurement systems support continuous improvement while others reinforce compliance. Previous research further emphasizes that learning-oriented measurement depends on locally meaningful indicators, professional involvement and supportive governance conditions [1, 7–9]. Overall, this body of research suggests that defining “what matters” in monitoring is not merely a technical task, but an organizational and political process shaped by competing governing logics [2, 10].

Complex healthcare systems are characterized by the interdependence of multiple actors, organizations and processes [11]. Complexity arises not only from the number of components involved, but also from the diversity and interdependence of the relationships between them. These interdependencies give rise to system-level properties such as non-linear dynamics and emergent outcomes that cannot be fully understood by analysing individual components in isolation. Healthcare systems are therefore often described as complex adaptive systems in which reforms, governance mechanisms and measurement practices interact with existing organizational structures and professional relationships in ways that may produce unintended or difficult-to-predict effects [12]. From this perspective, attempts to govern reforms through measurement are likely to encounter structural constraints that arise from interdependencies.

Many contemporary healthcare system reforms seek to address this complexity by strengthening co-ordination across services and placing greater emphasis on person-centred care [13–15], particularly in response to ageing populations and increasing multi-morbidity [16]. Person-centred care broadly emphasizes partnerships between patients and professionals, shared decision-making, co-ordination of care across organizational boundaries and recognition of patient capabilities [17–19]. While considerable research has focused on conceptualizing and measuring person-centred care at the clinical level [20–24], policy analyses suggest that translating these principles into operational practices remains challenging [25–27].

Turning such reform ambitions into practice requires appropriate measurement systems for monitoring progress and assessing whether desired transformations are taking place [28]. Although previous research has examined the design, implementation and effects of performance measurement in complex healthcare systems [2, 29], this work has primarily focused on the technical properties of indicators (e.g. availability, quality and comparability) or their impact on organizational performance. Less attention has been paid to how follow-up priorities – understood here as decisions about what should be monitored in reform governance – are defined when large-scale reforms are translated into local governance contexts [30–32]. In practice, stakeholders must define what should be monitored while operating within fragmented data infrastructures, competing institutional expectations and varying levels of data literacy [29–31, 33]. Municipal stakeholders in particular often sit at the interface between national reform ambitions and the practical constraints of local service delivery. In Sweden, these challenges are particularly visible in the ongoing transition toward “Close Care” (in Swedish: Nära vård), a national reform aiming to strengthen primary care and shift the healthcare system away from hospital-centred models toward more person-centred, co-ordinated and preventive services [34, 35].

The Close Care reform is closely aligned with the conceptual foundations of integrated care [14, 26], as both emphasize person-centredness, continuity and co-ordination across organizational boundaries, as well as the reduction of fragmented service delivery. However, the concepts are not synonymous. While integrated care constitutes a broader international umbrella concept referring to the design and delivery of co-ordinated services around people’s needs across time, sectors and levels of care, Close Care represents a specifically Swedish transformation agenda. It operationalizes similar principles through a stronger emphasis on accessibility, prevention, partnership with patients and families and care delivered as close as possible to the person’s everyday life context. As such, Close Care can be understood as a national policy and practice expression of integrated person-centred care, with a distinct reform orientation towards system-wide rebalancing of the healthcare system.

Translating this reform into practice has proven challenging due to resource constraints and misalignments between national ambitions and local service realities [36]. The reform requires closer collaboration between regional health services and municipal healthcare and social care, making meaningful follow-up dependent on indicators that capture developments across organizational boundaries. Defining such follow-up priorities has therefore become an important governance task for regional and municipal stakeholder [37]. Previous research also indicates that the reform is often perceived as conceptually broad, creating uncertainty about how its ambitions should be operationalized and translated into measurable objectives [38].

Developing such measurement systems has, however, proven difficult. Although Sweden is often perceived as having highly developed health data infrastructures because of its use of personal identity numbers and national quality registries, these systems were largely developed to monitor diagnoses, treatments and the performance of individual providers rather than integrated, person-centred care across organizational boundaries [39]. Health and social care data are furthermore collected and managed within separate organizations and cannot routinely be linked across organizational boundaries, while data for municipal health and social care remain considerably less developed than those available for regional healthcare. Municipalities also use multiple information systems, making standardized data extraction difficult [37].

Several national agencies have highlighted these challenges in their evaluations of the reform [39–41]. The Swedish Agency for Health and Care Services Analysis has concluded that broad reform ambitions, fragmented governance and limitations of existing data make it difficult to assess progress towards the reform’s overall objectives [40, 42, 43]. The National Board of Health and Welfare has compiled national indicators and key figures to support follow-up of the reform [39, 44]. However, these indicators largely rely on existing data sources, which were not designed to evaluate co-ordinated, person-centred care across organizational boundaries.

Crucially, current measurement systems were not designed to capture many of the reform’s defining ambitions, leaving important dimensions of the transformation largely invisible. For example, no national patient-reported experience measures capture patients’ experiences of municipal primary care. Existing national patient surveys are primarily organized around individual healthcare providers and focus on patients’ most recent healthcare encounter rather than their overall experiences of care delivered across multiple organizations and professions [37]. Consequently, current measurement systems provide only limited support for evaluating the broader ambitions of Close Care or guiding regional and local governance. This mismatch between the ambitions of the reform and the design of existing measurement systems prompted the establishment of a commission to develop a regional follow-up model for Close Care in Västra Götaland County, providing the empirical setting for the present study.

### Conceptual framing: governing “what matters” in complex healthcare systems

To conceptualize these challenges, this study draws on broader debates on how indicators function in the governance of complex public sector systems. Rather than treating indicators as neutral tools for monitoring performance, this study approaches them as governance instruments that shape priorities, accountability, relationships and possibilities for action [5, 45].

The analysis is informed by three complementary strands of literature. First, research on performance measurement and performance management highlights how indicators function both as governance tools and as sources of unintended consequences such as gaming, tunnel vision and symbolic compliance [2, 4, 5]. Second, studies of complex healthcare systems emphasize how governance interventions interact with existing institutional arrangements and professional practices in ways that may produce unpredictable outcomes [10, 11]. Third, scholarship in the sociology of quantification and audit cultures highlights that indicators are produced through social and organizational processes in which actors negotiate what should count as relevant knowledge [45, 46].

Taken together, these perspectives suggest that defining follow-up priorities is not merely a technical exercise of indicator selection but a governance process through which actors negotiate what should become measurable. Against this background, this study examines how municipal stakeholders negotiate what should be measured when performance indicators are introduced as governance tools in a large-scale healthcare system reform. Specifically, it investigates the governance process through which follow-up priorities are defined before indicators are developed, implemented and used.

## Methods

This study was designed using an interpretive descriptive approach, as outlined by Thorne [47]. Interpretive description is a qualitative methodology developed for applied health research, aiming to generate analytically informed insights that are directly relevant for practice and policy rather than developing formal theory. Within this approach, the analysis is guided by an iterative and interpretive process that identifies patterns and relationships while remaining grounded in participants’ accounts and the applied context of the study. In this study, interpretive description provided an overarching methodological framework for examining how stakeholders define and negotiate follow-up priorities in the context of a complex healthcare system reform.

Guided by this approach, we conducted workshops with municipal stakeholders to explore how follow-up priorities for the Close Care reform are defined in practice. Participants were asked to identify existing indicators, articulate gaps in available information and propose additional measures to support a regional monitoring model.

### Setting

Sweden’s healthcare system is highly decentralized. National authorities set overarching legislation and policy, while 21 regions and 290 municipalities are responsible for financing, planning and delivering health services [48]. The system is largely tax-funded, with regions accounting for approximately three quarters of total healthcare expenditure and municipalities for the remaining one quarter. [49]

At the regional level, healthcare is organized into primary and specialized care. Regional primary care centres provide first-contact services such as medical consultations, preventive care and rehabilitation and refer patients to specialized services when necessary [48].

Municipalities are responsible for healthcare delivered in nursing homes and home-based healthcare for individuals with frequent care needs who, due to illness or disability, cannot easily access primary care centres. Municipal healthcare is primarily provided by registered nurses, physiotherapists and occupational therapists, while physicians are employed by the regions. Most tasks are, however, delegated to non-registered professionals, such as assistant nurses and care assistants employed in social care services. Many patients receiving municipal healthcare also receive assistance with daily living provided by social care services [50, 51]. The work of licensed healthcare professionals and social care staff is regulated by different legislation [52]. This institutional division of responsibilities creates a system in which co-ordination across organizational boundaries is essential.

### Workshops

The empirical data in this study were collected as part of a commission from the political co-ordinating body in Västra Götaland County, a group that provides recommendations for political decision-making concerning healthcare in all 49 municipalities in the county and the Västra Götaland Region [53]. The first author was one of two project leaders of the commission whose aim was to develop a regional monitoring model for the Close Care reform. This dual role provided unique access to an ongoing policy process and positioned the first author within the governance process being studied. In the role as project leader, the first author, in dialogue with a working group consisting of regional and municipal representatives, was responsible for developing the commission’s project plan and designing the workshop process, including the questions around which participants were invited to reflect. The project plan was subsequently endorsed by the political co-ordinating body without revision. The research questions and analytical approach were developed within the research project. The empirical material was therefore generated within a setting that the first author had also participated in shaping.

The commission organized a series of stakeholder workshops to support the development of a regional follow-up model for the Close Care reform. From a research perspective, these workshops provided a suitable empirical setting for examining how follow-up priorities were collectively negotiated among stakeholders rather than expressed as individual opinions. Workshops are structured settings where participants collectively engage in knowledge building, creative problem-solving and idea generation around a specific question [54]. Their interactive format made it possible to observe how participants jointly negotiated what should be measured, why it should be measured and how measurement could support governance.

Between January and March 2025, nine workshops were conducted with politicians, first- and second-line managers, civil servants and healthcare and social care professionals from the 49 municipalities in Västra Götaland County. The interactive format enabled participants from different organizational roles to reflect on monitoring needs and constraints in relation to one another’s perspectives. This made it possible to observe not only which indicators were proposed, but also how participants reasoned about feasibility, underlying values and the governance implications of different approaches to monitoring.

Although the workshops formed part of the commission’s work to develop a regional follow-up model for the Close Care reform, the research project used the workshop discussions as empirical material to investigate broader questions concerning governance and the conditions required for meaningful measurement in complex healthcare systems. The workshops thus served both as part of an ongoing governance process and as the empirical setting for the research. The study was not designed as action research or participatory research; however, the researcher’s position within the commission meant that the empirical setting was not independent of the researcher’s involvement. The analytical focus of the study was defined by the research questions rather than by the commission’s policy objectives, and the analysis was not undertaken to evaluate the commission or determine the content of the regional follow-up model.

Invitations were distributed through the commission’s working group via established professional networks, including directors of social services, municipal healthcare and elder care managers, politicians involved in regional healthcare and social care governance groups and municipal chief nurses and rehabilitation managers. These recipients were asked to disseminate the invitation further within their organizations. As distribution relied on existing professional networks, the research team did not have full control over its reach. Participation was voluntary and open to all individuals who registered their interest through a digital registration form. All who registered were offered a place in the workshops. Because the workshops formed part of a regional initiative to develop a follow-up model for the Close Care reform, they primarily attracted stakeholders involved in governance, implementation and strategic development. Although the invitation encouraged participation from strategic, managerial and front-line levels, the final sample included only two front-line care staff (Table 1), reflecting the governance focus of the workshops rather than the composition of the broader healthcare workforce.

**Table 1:** Number of participants in workshops by organization and role

	Workshop form	Politicians	Second-line managers	First-line managers	Civil servants	Care staff	Total
Municipal association 1	Digital	12					12
	Digital		10	2	14		26
Municipal association 2	Digital	2	4	1	5		12
	Digital			4	1	2	7
Municipal association 3	Digital		26		1		27
	Digital			16	20		36
	Physical	16					16
Municipal association 4	Physical	11	17				28
	Digital		5	4	18		27
Total	9	41	62	27	59	2	191

Second-line managers refer to managers who supervise other managers. First-line managers refer to managers who supervise staff without managerial responsibilities

Two or three workshops were held in each of the four municipal associations in which all 49 municipalities in the county are organized. Two workshops were conducted physically and the remainder digitally via Microsoft Teams. Participation ranged from 7 to 37 persons per workshop, with a total of 191 individuals taking part (Table 1). Four of the workshops were led by the first author, and the rest by the other project leader.

A preparatory document was distributed to all participants prior to each workshop. This material described the goals of the Close Care reform and previous monitoring practices of the reform at national and regional levels. Questions to be discussed at the workshops were circulated in advance, and participants were encouraged to prepare by discussing them with colleagues. The questions were:

(1) Which existing indicators should be included in a regional monitoring model?

(2) What information is missing for monitoring the Close Care reform?

(3) Which new indicators should be included in a regional monitoring model?

Each workshop followed the same structure: a short introductory presentation about the Close Care reform and current monitoring practices, followed by small group discussions (4–11 participants per group) focusing on the three questions. Group discussions lasted 30–45 min and were not formally moderated, although project leaders visited groups to clarify tasks when necessary. Documentation was collected through post-it notes (physical workshops) and digital post-it-notes (digital workshops). After discussions in smaller groups, participants reconvened in plenary to share key insights. The workshop structure ensured that discussions addressed both existing monitoring practices and potential future indicators.

All group and plenary discussions at the digital workshops were recorded and subsequently transcribed verbatim using an artificial intelligence (AI)-assisted transcription tool. The transcripts were reviewed by the first author to correct potential transcription errors and ensure accuracy. At physical workshops, one person in each group was asked to take notes.

Information about recording and subsequent use of the material, both for the development of a regional monitoring model and for academic research, was included in the invitation. The participants were also given oral information about the study at the beginning of each workshop. They consented to the recorded discussions and notes being used as data in the research study when they registered for the workshop.

### Data analysis

Consistent with an interpretive descriptive approach, the empirical material was analysed using an abductive thematic strategy. This approach allows for an iterative movement between empirical data, contextual knowledge and theoretical perspectives to develop practice-relevant analytical insights. In this study, abduction was understood as an iterative movement between empirical observations, sensitizing concepts derived from the reform framework and analytically informed interpretations of the conditions shaping measurement practices [55]. Thematic analysis was used to identify patterns across the dataset [56, 57]. The analysis began with the conceptual vocabulary articulated in the regional roadmap for Close Care [58], which provided the policy framework within which the workshops had been organized. Concepts including person-centredness, continuity, co-ordination, collaboration, individual care plans, designated care contacts, trust, digitalization, accessibility, outpatient care, mobile teams, self-care, self-monitoring and health promotion functioned as sensitizing concepts during the early stages of analysis. We examined how participants referred to these reform concepts when proposing indicators, describing information gaps or discussing challenges related to monitoring the reform. At the same time, the analysis was informed by the theoretical framing presented in the “Background” section, particularly literature on complex healthcare systems, performance measurement and governance and the sociology of quantification and audit cultures. Rather than applying these perspectives as fixed analytical categories, they functioned as sensitizing lenses that guided interpretation of the empirical material.

Through iterative coding and comparison across the dataset, patterns across the empirical material were progressively interpreted in relation to conditions shaping the feasibility of governing reform ambitions through measurement. Suggested indicators and discussions of follow-up priorities were examined not only in terms of their substantive content but also in relation to broader issues concerning what should be measured, why measurement should be undertaken, whose purposes it should serve, what could realistically be measured and how available data could be interpreted and used within governance processes. This analytical process ultimately informed the development of the four interrelated conditions presented in the “Results” section.

Three complementary sources of empirical material were analysed: transcripts of group discussions from the digital workshops, written notes from the physical workshops and digital and physical post-it notes documenting group reflections. All post-it notes were collated into a single Microsoft Excel file. Each comment related to one of the three discussion questions was entered into separate sheets, yielding 125 entries for question 1, 85 for question 2 and 155 for question 3. Duplicate statements were noted. The written data were read repeatedly to achieve familiarity, after which entries were tentatively grouped under preliminary thematic labels. These groupings were iteratively refined into broader patterns.

For the transcripts of group discussions, the first author conducted the primary analysis by identifying segments relating to reform concepts as well as recurring issues concerning measurement feasibility, data availability and governance purposes. Initial analytical work was undertaken in dialogue with the co-project leader of the commission. To support critical reflection and enhance analytical credibility, preliminary interpretations and thematic structures were subsequently discussed with another member of the working group, who was familiar with the empirical material and provided feedback on emerging patterns. Later in the process, interpretations and thematic structures were also discussed with the wider working group. The analysis was thus conducted as an iterative and collaborative interpretive process that drew on both research perspectives and participants’ contextual knowledge of the ongoing governance process, while the first author retained overall responsibility for the analytical decisions and thematic structure. Although the co-authors did not have direct access to the full dataset, E.W., H.W. and A.W. contributed to the analytical process by reviewing and commenting on drafts for a Swedish-language stakeholder report based on the same material [59]. Through these discussions, interpretations were challenged, clarified and refined. The final analytical structure was agreed upon by all authors. Illustrative quotations were selected to represent key patterns identified in the analysis. Throughout the process, the analysis focused not only on the indicators suggested by participants but also on how stakeholders reasoned about; what should be measured, why it should be measured, for whom it should be measured and under what conditions could measurement meaningfully support governance of the reform.

Reflexivity was considered throughout the research process, particularly given the first author’s dual role as both project leader of the policy commission and researcher. This position provided contextual knowledge and sustained access to the governance process, while also meaning that the first author had participated in shaping the workshop setting in which the empirical material was generated. Reflexivity therefore concerned not only how prior knowledge and relationships with participants might influence interpretation, but also how the researcher’s position contributed to the conditions under which particular questions, discussions and insights emerged.

Drawing on Thorne’s guidance regarding interpretive authority [47], the first author continuously examined these influences throughout the analysis. Initial interpretations were repeatedly questioned, alternative explanations actively explored and emerging analytical patterns assessed against the full empirical material before being developed into broader interpretations. Interpretations and analytical decisions were also revisited through discussions with the co-project leader, the research group and the working group consisting of regional and municipal representatives. These discussions helped identify alternative explanations, test the plausibility of emerging interpretations and relate the findings to practical governance contexts. The use of multiple empirical sources and collaborative interpretation further strengthened the credibility and analytical rigour of the study, while the first author retained interpretative responsibility for the final analysis.

## Results

Although the workshops were initially organized around domains articulated in the regional roadmap for Close Care [58], the analysis did not primarily reproduce these domains. Rather than focusing on specific indicators, participants’ discussions repeatedly returned to broader questions concerning why measurement should be undertaken, what should be meaningfully monitored, how measurement should function within governance and the conditions required for measurement to support reform implementation.

Four closely related empirical patterns were identified:

(1) Negotiating why, for whom, and how measurement should be used.

(2) Lack of shared understanding of the reform and what should be monitored.

(3) Infrastructural constraints shaping what can be measured.

(4) Interpreting and making sense of available data.

Through abductive interpretation, the empirical findings were synthesized into four interrelated conditions shaping the feasibility of measurement-based governance. These comprised normative conditions concerning why measurement is undertaken and whose interests it is intended to serve; conceptual conditions concerning what is to be measured; infrastructural conditions concerning the availability and organization of data; and epistemic conditions concerning how measurement results are interpreted and used. Together, these four conditions provide a conceptual framework for understanding how governance through measurement is shaped in complex healthcare system reforms.

### Normative conditions: negotiating why, for whom and how measurement should be used

The first pattern concerns how participants negotiated the purpose of measurement and its role within the governance system. Across workshops, participants expressed differing expectations regarding what monitoring should achieve and how it should function within the governance system. Some emphasized follow-up as a tool for quality improvement and learning, while others highlighted its role in accountability and oversight. While external reporting and benchmarking were acknowledged as important, several participants expressed concern that publicly reported indicators could be misinterpreted when removed from their organizational context. As one participant noted:

“These are public figures where we are compared nationally, and they end up in the hands of our politicians, who draw conclusions from them that are completely wrong. And I don’t want that.” (second-line manager).

Others stressed that data should primarily serve as a basis for reflection, dialogue and local improvement rather than as instruments of control. In these discussions, participants emphasized that meaningful follow-up requires opportunities for collective reflection on data rather than solely the production of indicators.

Several participants also noted that defining indicators is not a trivial task, as “what is measured is what people tend to work towards.” As one participant explained:

“The indicators need to help move us in the direction we want to go, not simply reflect what happens to be available and easy to measure.” (civil servant).

Some participants advocated for a limited number of clear and stable indicators that could provide strategic overview and political accountability. According to these participants, follow-up should enable comparability over time and across organizations, support high-level decision-making and offer transparency regarding progress. A concise indicator set was also seen as necessary to avoid reporting fatigue and to maintain focus on a manageable number of goals.

Others emphasized the need for more comprehensive and nuanced measurement frameworks capable of capturing the complexity of person-centred and co-ordinated care. In these discussions, concerns were raised that overly simplified indicators risked obscuring important qualitative dimensions, particularly those related to relational continuity, collaboration and patients’ experiences. Participants noted that some of the most central ambitions of the reform – such as trust, co-creation and holistic support – resist straightforward quantification.

### Conceptual conditions: lack of shared understanding of the reform and what should be monitored

The second pattern concerns conceptual conditions related to how the reform is understood and what is expected to change. Across workshops, participants repeatedly returned to questions about what, more precisely, was supposed to change in the reform. Central ambitions in the regional roadmap [58] – such as shifting care from hospital to primary care, strengthening continuity or increasing prevention – were described as open to multiple interpretations. For example, participants asked whether a shift from inpatient to outpatient care implied reducing hospital beds, increasing visits in regional primary care, expanding municipal home-based services or some combination of these. In several discussions, these ambiguities were further complicated by parallel national initiatives promoting increased hospital capacity, thereby introducing potentially conflicting policy signals. Some participants also described misunderstandings between organizations regarding how care is delivered in municipal settings:

“This is actually quite a common misunderstanding in our dialogue with the region. They assume that healthcare interventions are carried out by nurses. But every time we explain that it’s not nurses providing everyday home-care services, at best it is assistant nurses working under delegation.” (first-line manager).

Participants frequently linked this conceptual fluidity to difficulties in determining what should be measured and how follow-up priorities should be defined. As one participant articulated:

“If we’re going to discuss what indicators we should use to determine whether we are achieving the transformation [the Close Care reform] or not, then we first need to know what we actually mean by the transformation. And I think we struggle quite a lot with that, it’s somewhat vague, at least from the perspective of those of us working in the organizations.” (care staff).

Participants rarely positioned themselves as active drivers of the reform. Instead, the reform was often described as externally initiated – something that affected local organizations rather than a process locally initiated and defined. Discussions therefore frequently reflected a perception that the goals of the reform had been formulated elsewhere and had not yet been fully translated into the local organizational context.

### Infrastructural conditions: constraints on what can be measured

The third pattern concerns infrastructural conditions shaping what can be measured and how data can be used. While conceptual ambiguity complicated agreement on what should be monitored, participants also described substantial infrastructural constraints that limited what could realistically be measured. These constraints were not only described as practical barriers but also shaped how participants prioritized and negotiated between potential indicators.

Participants proposed numerous indicators intended to capture reform ambitions related to person-centredness, co-ordination, preventive work, team-based care and quality of collaboration. However, many of these suggested indicators were not currently available in existing datasets, were only partially recorded or were dispersed across multiple systems, making them difficult to retrieve or combine. As a result, discussions often shifted from what would ideally be meaningful to monitor toward what was practically possible to measure within existing infrastructures.

Data quality was another recurrent concern. Participants pointed to inconsistencies in registration practices, incomplete coding and variability in reporting standards. Even when relevant variables were formally collected, doubts were raised regarding their reliability and comparability. As one participant expressed, “we collect a great deal of data, but whether it tells us what we need to know is another matter.”

Challenges were discussed in relation to municipal healthcare, where individual-level data infrastructures were perceived as less developed compared with regional healthcare systems. Participants frequently described fragmented information systems as a major barrier to co-ordinated follow-up. As one participant explained:

“There may be continuity between people, but not between the systems. Are there ways to monitor so we can see whether our systems actually support our work? People working in the same workplace can’t even access each other’s systems. There are so many technical challenges.” (second-line manager).

Participants also noted that several desirable indicators – such as outcomes of co-ordinated individual care plans, continuity across providers or the patient’s trajectory across organizational boundaries – would require data integration across municipalities and regions. Such integration was often described as technically difficult or organizationally unfeasible within current system architectures. Participants further pointed out that current legislation in Sweden does not allow linkage of individual-level data across organizational boundaries between municipalities and regions, limiting the possibility of developing integrated indicators that capture patient trajectories across the system. This created a recurring tension between ambitions to capture patient trajectories across organizational boundaries and the practical limitations of existing data systems, often resulting in more fragmented or proxy measures.

### Epistemic conditions: interpreting and making sense of available data

The fourth pattern concerns epistemic conditions related to stakeholders’ awareness and interpretation of existing data. Even where relevant indicators formally existed, participants often expressed uncertainty about how these data should be interpreted or used for decision-making and quality improvement.

Several participants also questioned whether existing measurement approaches were suitable for municipal care contexts, where many patients receive care until the end of life rather than with the expectation of recovery. One participant asked:

“How do you measure quality in a residential care facility or in home-care services? It requires different approaches than the outcome measures typically used in hospitals.” (civil servant).

Participants also described situations where data were collected extensively but still difficult to interpret or use for improvement. As one participant pointed out:

“We’re good at collecting data, but when are we good at getting it out? We measure everything all the time, and still what we want to happen doesn’t happen.” (politician).

Participants often questioned whether numerical indicators alone could provide insight into whether care had improved or why certain patterns appeared in the data. Questions were raised about what specific metrics actually signified, how they should be contextualized and whether observed changes reflected meaningful improvements or merely shifts in registration practices. Several participants therefore called for strengthened analytical support at regional and inter-municipal levels, including recurring analytical reports or structured dialogue forums to jointly interpret data. Measurement alone was not perceived as sufficient; participants emphasized the need for forums where results could be collectively interpreted and discussed.

### Interacting conditions shaping measurement-based governance

**Fig. 1:**
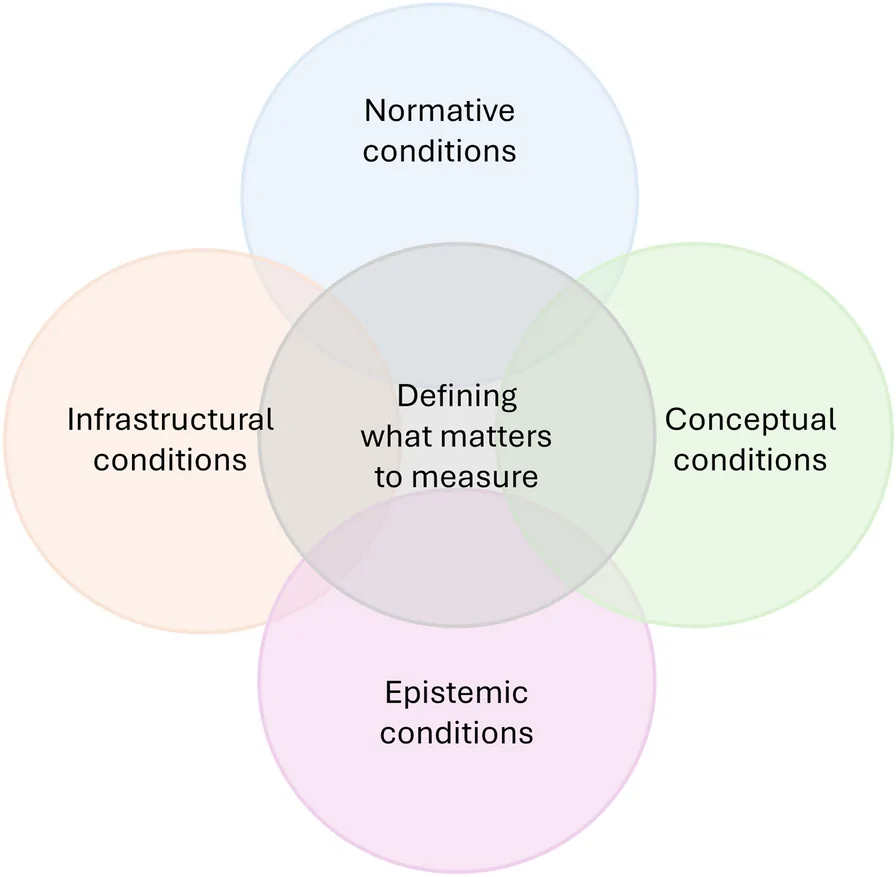
Mutually constitutive conditions shaping follow-up priorities in complex healthcare system reform governance

Taken together, the findings show that defining follow-up priorities involved the interaction of normative, conceptual, infrastructural and epistemic conditions (Fig. 1). These conditions did not operate as sequential stages or independent components. Normative negotiations about why, for whom and how measurement should be used were intertwined with understandings of the reform and its objectives. These conceptual understandings influenced which aspects of the reform were regarded as relevant to monitor, while existing data infrastructures constrained what could be measured in practice. Epistemic capacity, in turn, shaped how available data were interpreted and whether they were regarded as meaningful and useful. Figure 1 synthesizes these relationships as mutually constitutive conditions shaping the definition of what matters to measure.

## Discussion

This study examined how municipal stakeholders navigated the tension between reform ambitions and measurement feasibility when defining follow-up priorities in a large-scale healthcare system reform. The findings suggest that such priorities are not simply derived from predefined reform objectives or available data. Rather, they are negotiated through the interaction of competing purposes of measurement, interpretations of the reform, infrastructural possibilities and capacities for making sense of data.

The study contributes to research on governance through measurement by showing that this negotiation constitutes an early stage of the governance process. Before performance measures can influence organizational priorities and practices, actors must determine which aspects of the reform should become visible, how they can be represented through measurement and how the resulting information should be interpreted and used. The conceptual framework therefore shifts attention from indicator selection alone to the governance processes through which objects of measurement – and consequently, objects of governance – are constituted.

One implication concerns the competing purposes attributed to measurement. Defining follow-up priorities proved inseparable from negotiating whether measurement should primarily support accountability, organizational learning or strategic steering. These tensions are well documented in the performance management literature, where service-oriented ideals co-exist with control-oriented management systems [1, 2, 4, 60].

In this study, simplified and comparable indicators were often associated with accountability-oriented uses such as benchmarking, whereas more nuanced and context-sensitive measures were seen as necessary to capture relational aspects of person-centred and integrated care. These competing expectations illustrate that governance through measurement involves balancing different values and steering logics rather than choosing between them.

Under conditions of conceptual and infrastructural fragility, however, this balance becomes difficult to sustain. Measurement systems oriented primarily towards accountability risk narrowing attention to what is easily quantifiable, while purely learning-oriented approaches may lack the comparability required in publicly funded welfare systems.

Another implication concerns the instability of the reform’s governing object. Although participants broadly endorsed ambitions such as person-centredness, prevention and co-ordination, these concepts lacked shared and operationally precise definitions. This ambiguity is further reinforced by the reform’s grounding in the broader and inherently flexible concept of integrated care, which is translated into the Swedish context through the Close Care reform with additional emphases and policy aspirations. This aligns with previous research showing that broad policy concepts often remain interpretatively flexible during implementation [60–62].

Performance measurement presupposes relatively stable definitions of what constitutes quality, effectiveness or improvement [3, 63]. When reform objectives remain open to interpretation, measurement cannot be reduced to the technical operationalization of agreed goals. It also becomes a process of defining what the reform is understood to be and which forms of change should count as evidence of progress.

A further implication is that the practical feasibility of measurement depends on more than the formal availability of indicators. Even when reform ambitions could be translated into potential measures, stakeholders frequently encountered a mismatch between what they regarded as important to monitor and what current information systems were designed to capture. Moreover, fragmented information systems, inconsistent registration practices and limited interoperability meant that even existing data could rarely be integrated into coherent measures of reform progress. Similar challenges have been identified in research on social care data and healthcare system performance assessment, where fragmented infrastructure and limited standardization hinder the use of data for governance [8, 64, 65].

Finally, the availability of relevant data alone does not ensure governability. Governance through data depends on distributed epistemic capacity – that is, actors’ ability across organizational levels to access, interpret and collectively make sense of measurement results. Without such capacity, measurement risks becoming symbolic or compliance-oriented rather than informing action and improvement. These findings also extend established approaches for quality measurement, such as Donabedian’s framework [3], by suggesting that structural, process and outcome measures only become meaningful for governance when organizations possess both relevant data and the conditions for defining, interpreting and using them collectively.

Scholarship on performance measurement and public-sector governance has examined the expansion of audit, evaluation and performance measurement as mechanisms of public governance and highlighted the tensions they create between accountability, control and organizational learning [5, 66, 67]. Research on governance through numbers has further demonstrated that quantification is not a neutral representation of reality, but involves processes of classification and categorization through which particular phenomena become measurable, visible and governable [68–71]. Building on this literature, our study provides an empirical account of how municipal stakeholders negotiate what matters to measure within an ongoing healthcare system reform. The findings identify four interrelated conditions – normative, conceptual, infrastructural and epistemic – that shape these negotiations. They demonstrate how competing purposes of measurement, interpretations of reform objectives, existing data infrastructures and capacities for collective interpretation interact in defining follow-up priorities. In doing so, the study makes explicit how the negotiation of measurement priorities constitutes a governance process in its own right.

A central implication is that the tensions identified in this study should not be understood as implementation failures or resistance to measurement. Rather, participants repeatedly described organizational changes – including improved data infrastructures, interoperability, governance arrangements and interpretive capacity – as pre-requisites for meaningful measurement. The challenges therefore reflect structural features of governing complex healthcare systems rather than shortcomings in local implementation.

From this perspective, negotiating “what matters” is a governance process through which competing understandings of accountability, co-ordination, improvement and legitimacy are enacted. The challenge is therefore not merely to identify technically valid measures, but to create arrangements through which reform priorities can be clarified, represented in data and interpreted collectively. Measurement is consequently not only a means of documenting or steering reform; it also participates in constituting what the reform is understood to be and what forms of change become governable.

### Governance implications

The findings suggest that municipalities should approach follow-up as a governance activity rather than reducing it to a technical exercise in indicator development. Developing meaningful approaches to follow-up therefore requires explicit dialogue about why measurement is undertaken, whose interests it should serve and how reform ambitions should be interpreted locally. Shared understandings of reform objectives are a pre-requisite for deciding what should be measured and why.

The findings suggest that meaningful follow-up requires both stronger interoperability and the collection of new types of data. While improved integration of existing information systems is essential, municipalities also need measurement systems capable of capturing central reform ambitions, including person-centred care, continuity and co-ordination across organizational boundaries. This will require not only better integration of existing data but also the development of new forms of measurement, as current systems were largely designed to monitor diagnoses, treatments and activities within individual organizations rather than integrated care processes. Measurement systems therefore need to evolve in parallel with the reform itself.

Finally, municipalities need institutional capacity for collective interpretation of measurement results. Data rarely speak for themselves. Their value depends on opportunities for collective interpretation and reflection. Structured dialogue forums, recurring analytical reviews and cross-organizational learning arenas are therefore as important as the indicators themselves because they enable measurement to inform shared understanding, decision-making and organizational learning.

Table 2 summarizes these governance implications by relating each condition to key governance challenges and potential areas for organizational development. The considerations should be understood as mutually reinforcing rather than as a sequence of discrete recommendations. These governance considerations do not imply that clarifying reform goals or strengthening data infrastructures alone can resolve competing priorities or political tensions; instead, they identify areas that may strengthen the conditions for meaningful measurement-based governance when developed together.

**Table 2:** Conditions shaping measurement-based governance in complex healthcare systems

Condition	Key challenge	Governance considerations
Normative	Tensions between accountability and learning purposes	Explicit reflection on the purposes, values and governance logics underpinning measurement
Conceptual	Ambiguity in reform goals; lack of shared understanding	Shared interpretation of reform objectives and governance priorities
Infrastructural	Fragmented data systems; limited interoperability	Development of interoperable data infrastructures, improved data quality and new forms of data collection
Epistemic	Limited capacity to interpret and use data	Capacity for collective interpretation and use of measurement results

### Strengths and limitations

This study benefits from broad participation across multiple organizational levels and professions within the municipal healthcare context. This enabled the identification of shared structural challenges rather than isolated organizational concerns. The workshop format fostered active engagement and provided a space for sharing experiences across organizational boundaries. The study also drew on multiple forms of empirical material – including recorded group discussions, workshop notes and post-it comments – which enabled triangulation of perspectives and strengthened the robustness and analytical depth of the analysis.

A further strength is that the study was conducted in direct connection with an ongoing regional reform process, enabling analysis of how stakeholders negotiate measurement priorities in a real policy setting rather than in a hypothetical or retrospective context. This proximity to an ongoing reform process provided access to discussions taking place while governance arrangements and monitoring practices were still being shaped.

However, several limitations should be considered. First, front-line care staff were under-represented, consistent with the governance focus of the workshops rather than the composition of the broader healthcare workforce. The findings should therefore be understood as representing the perspectives of actors involved in shaping governance, implementation and follow-up of the Close Care reform rather than the experiences of front-line professionals delivering care in everyday practice. Similarly, the study focused on municipal stakeholders responsible for translating national reform ambitions into local governance and practice. Consequently, the perspectives of national policymakers and agencies were not included and are therefore beyond the scope of this study. Future research should examine how national policymakers, regional actors and front-line professionals perceive these governance challenges and where their perspectives converge or diverge.

Second, the workshop format may have influenced the data, as group discussions can encourage consensus-oriented reflections and may limit the expression of dissenting or highly critical perspectives. However, the municipal-only composition of the groups appeared to facilitate open discussion of challenges and frustrations associated with the reform. Unlike many cross-sector forums, where municipal and regional actors jointly discuss the reform, these workshops provided a space in which municipal representatives could critically reflect on governance challenges from their own organizational perspective. The empirical material therefore contained substantial disagreement, uncertainty and critical reflection, although we acknowledge that some views may nevertheless have remained unexpressed.

Third, in the physical workshops, only written notes were available, which may have limited the level of detail captured from those discussions compared with the recorded sessions.

Fourth, the findings should be interpreted in light of the study context. The study was conducted in Västra Götaland County, Sweden’s largest region, encompassing municipalities that vary considerably in size, geography, organizational arrangements and demographic characteristics, thereby providing a broad range of governance contexts. Nevertheless, organizational structures, collaborative arrangements and policy contexts may differ across other Swedish regions and internationally. Rather than aiming for statistical generalization, this study provides analytical insights into governance challenges associated with integrated healthcare reforms.

A further consideration concerns the dual role of the first author as both project leader of the regional commission and researcher. This position provided unique access to an ongoing governance process and enabled the generation of empirically rich and policy-relevant data. At the same time, the roles cannot be understood as entirely separate. As project leader, the first author was involved in shaping the workshop agenda and the questions around which participants reflected, thereby contributing to the conditions under which the empirical material was generated. In this sense, the research has affinities with interventionist approaches in science and technology studies, which recognize that researchers may participate in shaping the practices and settings they study rather than remaining external observers [72, 73].

This proximity was therefore both analytically productive and a potential influence on the findings. It provided insight into an ongoing process in which measurement priorities were being negotiated, while also requiring reflexive attention to how the first author’s prior knowledge, relationships with participants and role in organizing the workshops could shape both the discussions and their subsequent interpretation. Throughout the analytical process, emerging interpretations were therefore critically examined, alternative explanations were actively sought, and preliminary analyses were discussed with the co-project leader, working group and co-authors. The dual role is thus understood not simply as a potential limitation to be controlled, but as part of the conditions through which the empirical material and analytical insights were produced.

Finally, as an exploratory qualitative study, the findings do not assess the effects of specific measurement systems but rather illuminate the conditions under which such systems are expected to operate. The study therefore contributes primarily analytical insights into how measurement priorities are negotiated and interpreted in governance contexts. Future research could examine how specific instruments and indicators are subsequently used in practice and how they influence decision-making, professional behaviour, quality improvement and organizational learning.

## Conclusions

By examining how follow-up priorities are negotiated in practice, this study shows that the feasibility of measurement-informed governance is shaped by the interaction of normative, conceptual, infrastructural and epistemic conditions. The findings provide empirical insight into how the negotiation of follow-up priorities shapes what becomes measurable and governable during healthcare system reform. This perspective highlights that measurement does not simply document reform progress but also shapes how reforms are understood, prioritized and governed.

Rather than viewing tensions between reform ambitions and measurement feasibility as implementation failures, the findings suggest that such tensions are inherent features of governing complex healthcare systems. Meaningful measurement depends on conditions that extend well beyond the optimization of indicators or data systems. Recognizing this may support more realistic expectations of what measurement can achieve by shifting attention from the technical optimization of indicators to the governance processes through which priorities are negotiated and reform is continuously shaped.

## Data Availability

The datasets generated and/or analysed during the current study are not publicly available to maintain participants’ confidentiality. However, they can be obtained from the corresponding author upon reasonable request.
